# Mechanical and Moisture Performance of Dense-Graded Cold-Recycled Asphalt with 60% RAP and Cement–Fly Ash Additives

**DOI:** 10.3390/ma19173751

**Published:** 2026-09-03

**Authors:** Abdul Qudoos Mallano, Naeem Aziz Memon, Antonio D’Andrea, Giuseppe Loprencipe, Laura Moretti

**Affiliations:** 1 Department of Civil Engineering, Mehran University of Engineering and Technology, Jamshoro 76062, Pakistan; naeemaziz@uitm.edu.my; 2 Faculty of Civil Engineering, Universiti Teknologi MARA, Shah Alam 40450, Selangor, Malaysia; 3 Department of Civil, Constructional and Environmental Engineering, Sapienza University of Rome, Via Eudossiana 18, 00184 Rome, Italy; antonio.dandrea@uniroma1.it (A.D.); laura.moretti@uniroma1.it (L.M.)

**Keywords:** cold-mix asphalt, reclaimed asphalt pavement, cement, Class C fly ash, Marshall stability, indirect tensile strength, moisture resistance

## Abstract

Emulsion-based cold recycling can reduce the demand for virgin aggregate and high-temperature asphalt production, but mixtures with high reclaimed asphalt pavement (RAP) contents often have limited early strength and moisture resistance. This study evaluated a dense-graded cold-mix system containing 0%, 60%, and 70% RAP, combined with a 2 × 3 additive matrix comprising cement (0.5% and 1.0%) and Class C fly ash (0.5%, 1.0%, and 1.5%). The 70% RAP blend fell outside the adopted dense-graded envelope, whereas the 60% blend satisfied that envelope and was therefore selected, not claimed as a universal optimum for the additive stage. Hot-mix asphalt was included only as a benchmark. Marshall stability, indirect tensile strength, Marshall quotient, tensile strength ratio, retained Marshall stability, and retained Marshall quotient were measured under the reported laboratory curing and conditioning procedures. Among the tested cold mixtures, the 60% RAP formulation containing 1% cement and 1% fly ash produced the highest measured stability (13.1 kN), dry indirect tensile strength (1.11 MPa), and Marshall quotient (6.89), with retained-property indices of approximately 87%. These short-term results indicate that gradation control and low-dose cementitious additions can improve strength development and moisture retention in the studied emulsion–RAP system. The contribution of the work is an integrated, formulation-level comparison of RAP screening and a cement–fly ash dosage matrix; the findings remain specific to the materials, gradation envelope, curing regime, and screening tests used.

## 1. Introduction

Cold-mix asphalt (CMA) is produced with an asphalt emulsion at or near ambient temperature and can reduce heating demand and virgin material consumption relative to conventional hot-mix production. Its environmental benefit is nevertheless project-specific and should be demonstrated through a defined life-cycle and economic assessment rather than inferred from processing temperature alone. Recent low-carbon binder research also shows that recycled material benefits depend on binder chemistry, additives, and aging response [1,2,3,4,5,6]. Reviews of cold mixtures and reclaimed asphalt pavement (RAP) identify emulsion breaking, curing, aggregate packing, and RAP variability as controlling factors [3,7,8,9,10,11,12,13,14,15,16].

RAP contributes mineral aggregate and aged binder, but the extent to which the aged binder is mobilized in a cold process is not necessarily equal to its measured total content. High RAP contents can therefore alter gradation, coating, compaction, air voids, and the balance between stiffness and flexibility [9,10,11,17,18,19,20,21,22,23,24,25,26,27,28,29]. Established cold-recycling frameworks—including Wirtgen, ARRA/FHWA, Austroads, and South African TG2 guidance—use project-specific gradation, binder, water, active filler, curing, and performance criteria and can permit very high RAP contents. In the present work, a dense HMA-type envelope was deliberately retained as a laboratory design constraint; consequently, the feasible RAP level found here cannot be generalized to other cold-recycling frameworks.

Cement and Class C fly ash are active cementitious constituents, not merely inert fillers. Cement can accelerate emulsion breaking and hydration-based strength development, while reactive fly ash can contribute secondary cementitious products when sufficient calcium, moisture, and curing are available [30,31,32,33,34,35,36,37,38,39,40,41,42,43]. These reactions may improve monotonic strength and moisture retention, but excessive hydraulic bonding can also increase stiffness and cracking susceptibility. Microstructural techniques such as scanning electron microscopy (SEM), Fourier-transform infrared spectroscopy (FTIR), and X-ray diffraction (XRD) are useful for verifying proposed reaction mechanisms; such measurements were not conducted in this study.

Previous studies have already evaluated cold mixtures with RAP contents above 60% and systems containing cement and fly ash [17,19,20,35,38,44]. Accordingly, the specific contribution of the present study is defined as follows: the same local material set and dense-graded design procedure were used to (i) screen 60% and 70% RAP blends, (ii) evaluate six distinct cement–Class C fly ash combinations at the selected 60% RAP level, and (iii) compare short-term mechanical and retained-property indicators with unmodified CMA and an HMA benchmark. The study does not establish a universal optimum RAP content or equivalence to long-term HMA performance.

## 2. Materials and Methods

### 2.1. Aggregates

**Figure 1:**
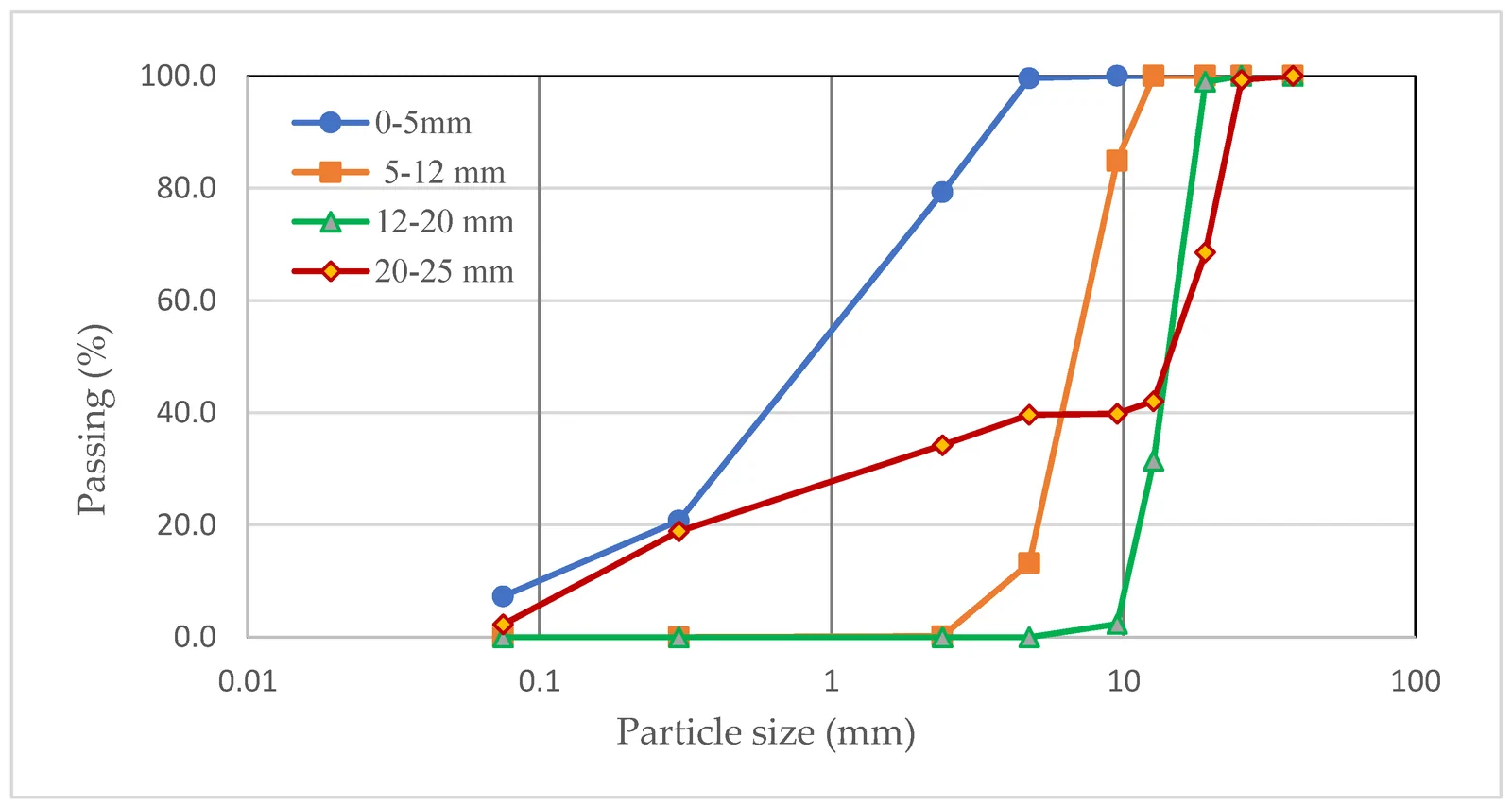
Gradation curves for all the four stockpile aggregates.

The crushed limestone aggregates were separated into coarse (5–25 mm) and fine (0–5 mm) fractions sourced from Noori-Abad quarry in Sindh, Pakistan, from four stockpiles (i.e., 20–25 mm, 12–20 mm, 5–12 mm, and 0–5 mm) whose physical properties are summarized in Table 1, while their corresponding gradation curves are illustrated and further discussed in Figure 1.

### 2.2. Bitumen

The bitumen used for the control HMA mixture was a 60/70 grade sourced from a refinery in Karachi, Pakistan, and its physical properties are illustrated in Table 2.

### 2.3. Bitumen Emulsion

The selection of emulsion for use in CRM depends on the aggregate’s surface characteristics. Aggregates with high silica content generally carry a negative surface charge and therefore bond effectively with positively charged cationic emulsions. In this study, high-silica aggregates were used with a cationic slow-setting emulsion (CSS-1h). The properties of the bitumen emulsion are in Table 3.

### 2.4. Reclaimed Asphalt Pavement

**Figure 2:**
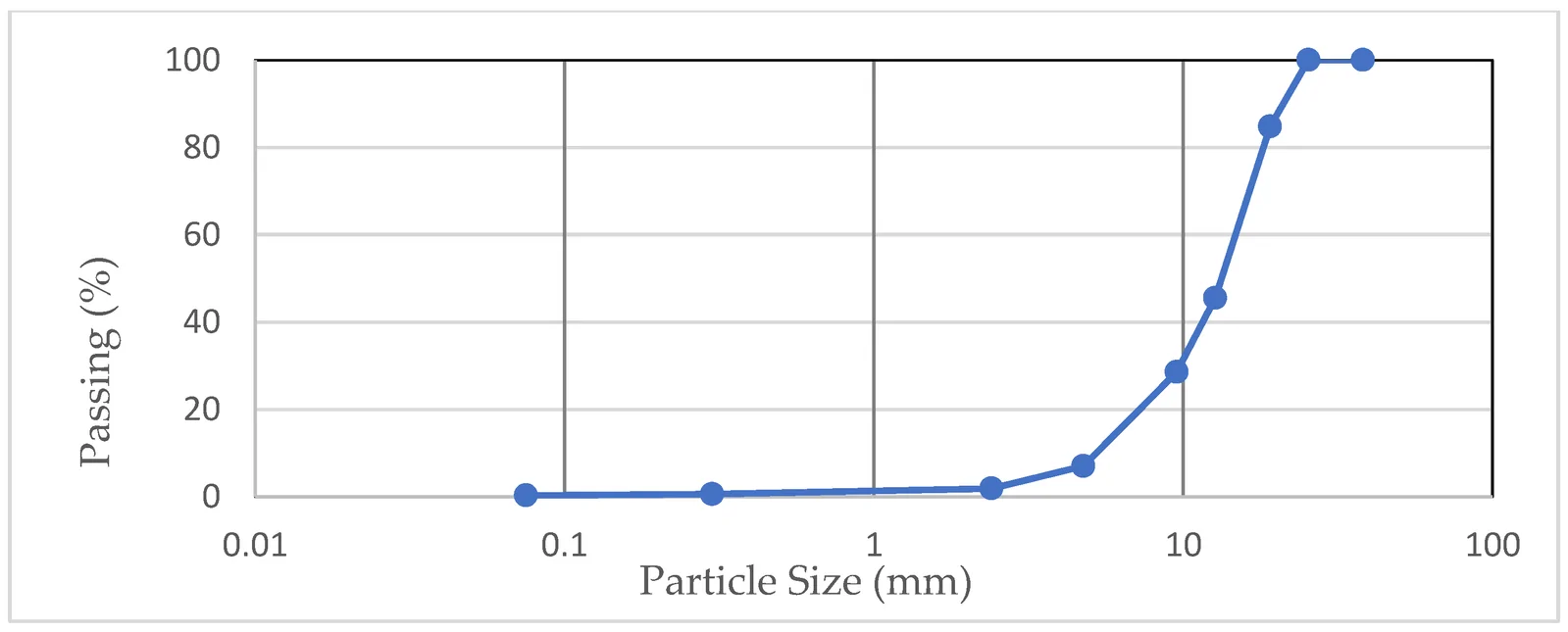
Sieve grading curve of as-received RAP.

RAP was collected from the M-9 motorway near the Jamshoro toll plaza in Pakistan, following pavement rehabilitation. Field observations indicated whitish aggregate surfaces consistent with binder stripping. Prior to gradation, oversized agglomerations were manually processed to a maximum particle size of 25 mm. Figure 2 presents the as-received RAP gradation (with the aged binder film intact), which was subsequently used as the basis for RAP–virgin aggregate blending calculations. RAP binder content was determined in accordance with AASHTO T164/ASTM D2172, followed by recovery of the extracted binder using a rotary evaporator per ASTM D5404. Binder content was calculated gravimetrically as the mass loss of the RAP sample (1200 g) following extraction, expressed as a percentage of the original mass. Three replicate determinations yielded 2.3%, 2.5%, and 2.7% (mean = 2.5 ± 0.2%). Extracted bitumen and RAP physical properties are provided in Table 4. The gradation envelope adopted corresponded to a 19.0 mm (3/4-inch) NMAS dense-graded gradation per the NHA Pakistan specification for dense-graded mixtures used in surface wearing course Class A, which is closely related to AASHTO M323 for designing HMA, as shown in Table 5. The objective of this selected envelope was to design a dense-graded surface course comparable to conventional HMA practices, rather than to adopt an established cold-recycling gradation framework, which typically permits looser gradation tolerances and correspondingly higher RAP contents. The 70% RAP blend failed the specification at the 3/4-inch, 3/8-inch, and No. 4 sieves, reflecting RAP’s inherently coarser mid-range gradation and the correspondingly reduced virgin fine-aggregate fraction available at higher RAP substitution levels (29% and 37% for the 70% and 60% RAP blends, respectively). The 60% RAP blend satisfied the envelope at all sieves, confirming that 60% RAP represents the highest feasible content within the adopted dense-graded HMA-type design framework; this constraint is acknowledged as a limitation of this study.

### 2.5. Cement and Class C Fly Ash Additives

In this study, the conventional aggregate mineral filler was partially replaced by ordinary Portland cement and Class C fly ash. These materials are described as active additives because they can affect emulsion breaking, hydration, stiffness development, moisture response, and cracking susceptibility. At the selected 60% RAP level, cement was used at 0.5% and 1.0% and fly ash at 0.5%, 1.0%, and 1.5% by mass of total dry aggregates. The physical and chemical properties of cement and fly ash are shown in Table 6 and Table 7, respectively.

### 2.6. Mix Design and Sample Preparation

**Figure 3:**
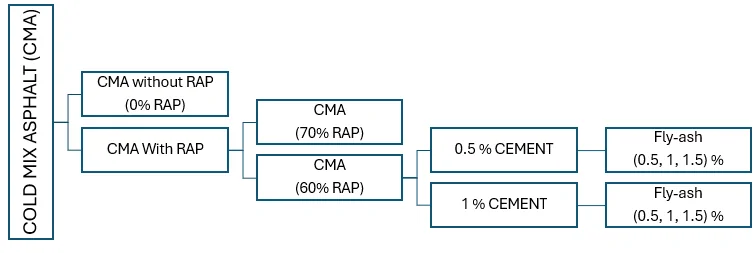
Design methodology.

Ten individual formulations were evaluated: one HMA benchmark, one CMA without RAP, two unmodified RAP screening blends, and six 60% RAP mixtures containing distinct cement–fly ash combinations (Figure 3 and Table 8).

### 2.7. Design and Sample Preparation of Mixtures

**Figure 4:**
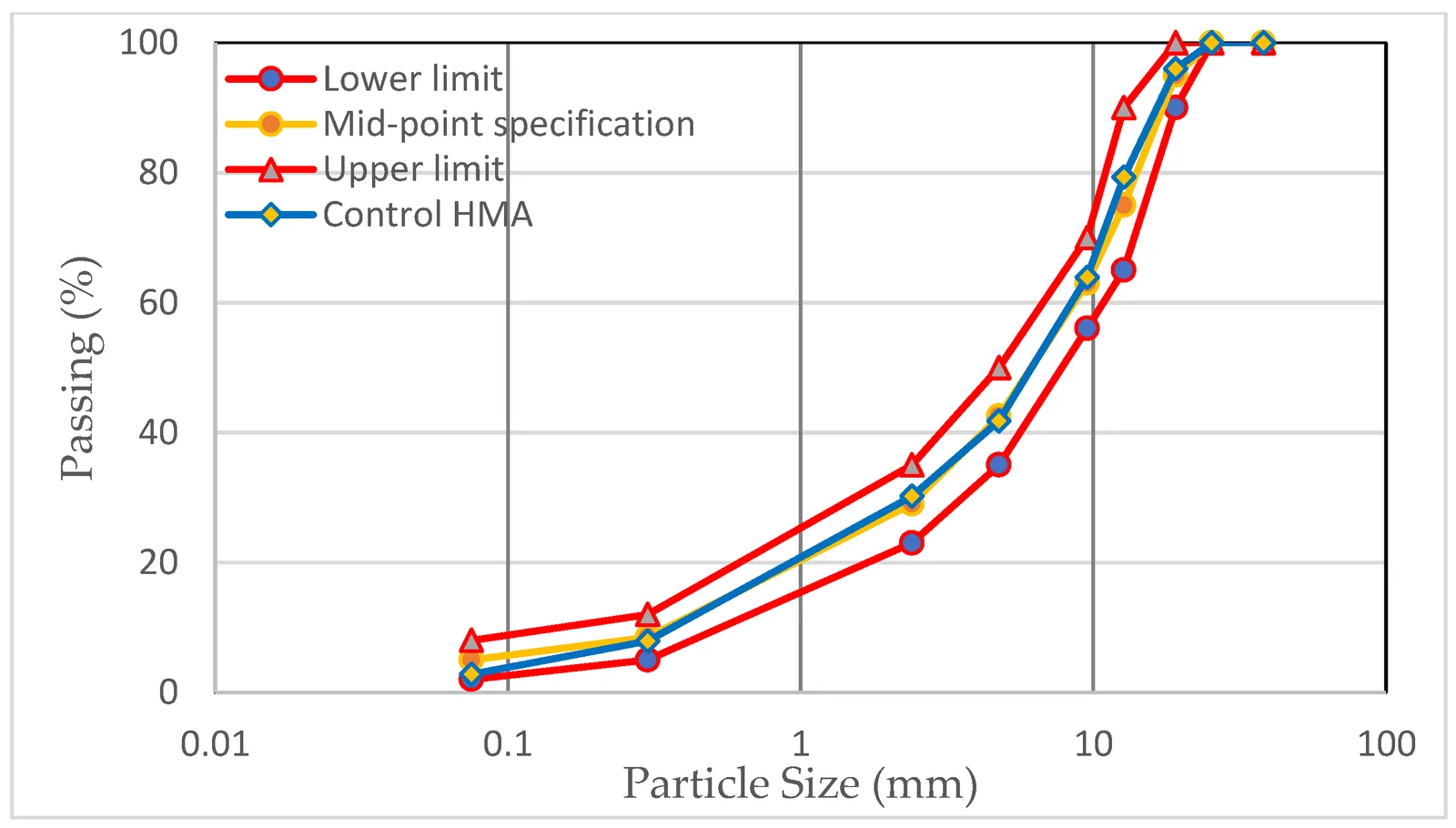
Gradation curve of the control HMA.

Mix0 was designed using a dense-graded Marshall procedure (Figure 4). The percentages of aggregate stockpiles (20–25 mm, 12–20 mm, 5–12 mm, and 0–5 mm) were selected as 12%, 20%, 30%, and 38%, respectively, after adjusting the binder %.

**Figure 5:**
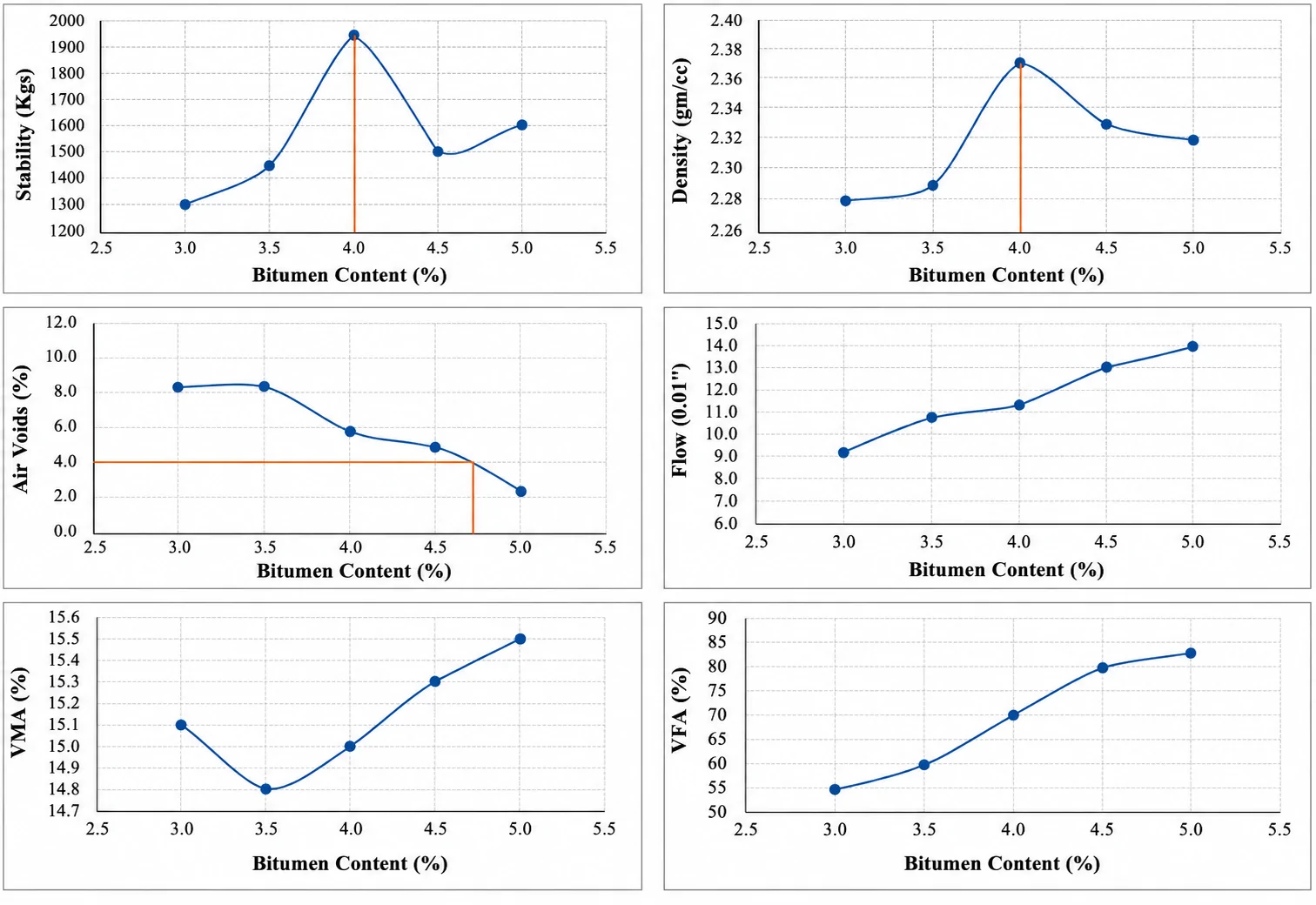
Properties of HMA.

Five bitumen contents (3.0%, 3.5%, 4.0%, 4.5%, and 5.0%) were tested with three replicate specimens at each content (15 specimens total). For each 1200 g specimen, materials were mixed at 154–160 °C, placed in a preheated 100 mm-diameter mold at 138–149 °C, and compacted with 75 blows per face. After 24 h at room temperature, specimens were tested for volumetric properties; specimens for stability and flow were conditioned at 60 °C for 1 h. The optimum bitumen content (OBC) was calculated as the average of the binder contents corresponding to maximum Marshall stability, maximum bulk density, and 4% air voids, which was found as 4.23%, as shown with orange lines in Figure 5 [37,45,46,47,48].

Mix1 was designed in accordance with the Asphalt Institute’s Manual Series No. 14 (MS-14, 1989) [49], using the same targeted gradation curve applied in the control HMA following the dense-graded mix protocol. For Mix2 and Mix3 incorporating 70% RAP and 60% RAP RCMA, separate gradation blends were developed. The blend containing 70% RAP did not comply with the specified gradation limits (Table 9), although some samples were prepared to verify the observations. In contrast, the 60% RAP blend satisfied all specification limits.

The initial residual asphalt content (IRAC) and initial emulsion content (IEC) were estimated using MS-14 [49]. Equations (1) and (2) were used for the control CMA, and Equation (3) adjusted the new binder demand for the RAP mixture following the guidelines in *Asphalt Institute Manual Series No. 20* (MS-20, 2007) [50]. Table 10 distinguishes the preliminary design estimates from the residual asphalt contents selected after the Marshall-based optimization sequence.

(1) IRAC = (0.05A + 0.1 B + 0.5C) × (0.7)

In Equation (1), A is the aggregate percentage retained on the 2.36 mm sieve, B is the percentage passing 2.36 mm and retained on 0.075 mm, C is the percentage passing 0.075 mm, and IRAC is expressed as a percentage of the total aggregate mass.

(2) IEC = (IRAC/X)

In Equation (2), X is the residual asphalt fraction of the emulsion (reported as approximately 60%); the remaining emulsion phase consists primarily of water and emulsifier.

(3) $$\mathrm{Pnb}=P-\frac{(100-r)\times Psb}{100}$$

In Equation (3), Pnb is the new residual asphalt to be added, Psb is the binder content measured in RAP, and r is the virgin aggregate proportion.

A 1200 g batch containing aggregate, RAP, additive, and pre-wetting water was dry-mixed; emulsion was then added and mixed for 2–3 min. Pre-wetting water was varied in 0.5 percentage-point increments. The optimum pre-wetting water content (OPWWC) was defined as the content producing more than 75% visually assessed coating.

Specimens prepared at the IEC and OPWWC were compacted with 75 blows per face. Water content was then varied in 1 percentage-point increments by air-drying, and dry density was measured. The optimum total liquid content (OTLC) for the considered mixes are reported in Table 10, and these values are defined at maximum dry density.

At the reported OTLC, the residual asphalt content was varied by ±1 percentage point in 0.5-point increments. CMA levels were 5.3%, 5.8%, 6.3%, 6.8%, and 7.3%; RCMA levels were 4.3%, 4.8%, 5.3%, 5.8%, and 6.3%. Three specimens per level remained in the mold for 24 h, were oven-cured at 60 °C for 48 h, and then conditioned for 24 h at room temperature before volumetric and mechanical testing. The optimum residual asphalt content (ORAC) was selected using the reported Marshall procedure. A complete formulation matrix of all mixes in percent by mass of total dry aggregates is presented in Table 11.

OPWWC, OTLC, and ORAC were determined using the unmodified 60% RCMA mixture (Mix3) and held constant across all cement–fly ash-modified formulations (Mix4 and Mix5). Given the relatively low active filler dosages evaluated (0.5–1.5% by mass), their influence on optimum liquid and binder demand was assumed to be limited; however, this was not independently verified through re-optimization and is acknowledged as a scope limitation of the study.

Table 11 presents the formulations of all mixes using the optimum values of OPnb and OTLC shown in Table 10, except for HMA. The required emulsion quantities for the control and RCMA were calculated by dividing OPnb values with X. Moreover, the required added-water quantities were obtained by subtracting used emulsion quantities from OTLC values.

### 2.8. Testing Methods

MS and flow were measured according to ASTM D6927 at 50.8 mm/min after the stated conditioning. Stability was the peak load and flow was the deformation at that load. The MQ was calculated as stability divided by flow and is used here only as a comparative stiffness/deformation screening index, not as a direct measure of field rutting performance.

The ITS of the mixtures (dry and wet) was evaluated following ASTM D6931 to assess the bonding capacity of the binder and the cracking resistance under dry and wet conditions. For dry ITS, Marshall samples were conditioned in a water bath at 25 °C for 2 h. A loading rate of 50 mm/min was applied diametrically until failure, and the peak load was recorded. For wet ITS, specimen conditioning followed ASTM D4867/D4867M prior to testing, with the peak load recorded at failure.

Durability was evaluated through multiple durability indices:

1. Moisture susceptibility was assessed by comparing wet and dry ITS values following ASTM D6931 and D4867/D4867M. The TSR was calculated using Equation (4):

(4) $$\mathrm{TSR}=\frac{wetITS}{dryITS}\times 100$$

2. RMS evaluates the effect of moisture on mixture stability. Dry stability was measured after conditioning the specimen for 30–40 min at 60 °C, whereas wet stability was measured after 24 h immersion at 60 °C (Equation (5)):

(5) $$\mathrm{RMS}=\frac{wet\;stability}{dry\;stability}\times 100$$

3. The RMQ compares MQ values before and after moisture conditioning (24 h at 60 °C) (Equation (6)):

(6) $$\mathrm{RMQ}=\frac{MQ\;after\;conditioning}{MQ\;before\;conditioning}\times 100$$

Statistical significance among mixtures was evaluated using ANOVA at a 95% confidence level, based on three replicate specimens per formulation. Three separate analyses were conducted: (i) a one-way ANOVA comparing unmodified CMA, 60% RCMA, and 70% RCMA to evaluate the effect of RAP content, with Tukey HSD post hoc comparison; (ii) a one-way ANOVA with Dunnett’s post hoc comparison, comparing unmodified 60% RCMA against six cement–fly ash-modified formulations, to evaluate the effect of active filler modification; and (iii) a two-way ANOVA on the six modified formulations, treating cement dosage (0.5%, 1.0%) and fly ash dosage (0.5%, 1.0%, and 1.5%) as independent factors, to distinguish their individual and interactive effects. For all analyses, differences were considered statistically significant at *p* < 0.05. The results for Marshall stability, indirect tensile strength, Marshall quotient, tensile strength ratio, retained Marshall stability, and retained Marshall quotient are presented in Section 3.7 (Table 12, Table 13 and Table 14).

## 3. Results and Discussion

In this section, two volumetric properties and three key mechanical properties—bulk density, voids in total mix (VTM), MS, ITS, and MQ—of all mix formulations are presented. HMA is treated as a benchmark; similarity in an individual index is not interpreted as equivalent overall pavement performance.

### 3.1. Bulk Density

**Figure 6:**
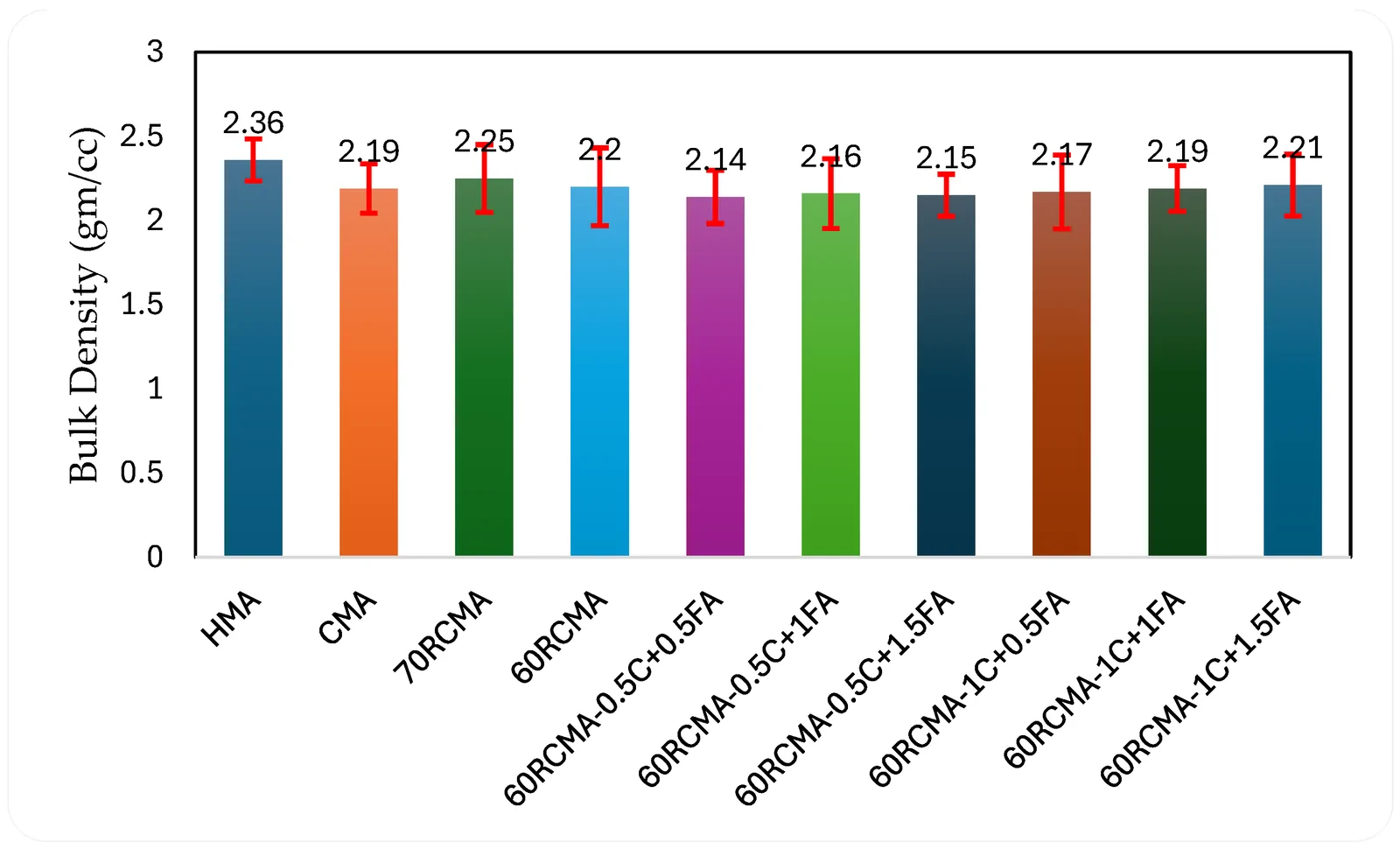
Bulk density results.

Figure 6 presents the bulk density for all mixtures. HMA achieved the highest density, 2.36 g/cc, due to superior compaction under hot-mix conditions. Initially, there was an initial density penalty upon the addition of cement and fly ash compared with unmodified 60RCMA. When the cement content was 0.5%, the densities were in the range of 2.14 to 2.16 g/cm^3^. However, adding more cement (0.5% to 1.0%) at all corresponding fly ash levels (0.5%, 1.0%, and 1.5%) was always found to increase mixture density, ranging from 2.14 to 2.17 g/cm^3^ at 0.5%, 2.16 to 2.19 g/cm^3^ at 1.0%, and 2.15 to 2.21 g/cm^3^ at 1.5% fly ash. For this reason, the 60RCMA-1C + 1.5FA formulation gave the highest density among the modified formulations and was very close to the density of the unmodified 60RCMA. These results show that increasing the cement dosage partially compensated for the volumetric drawback associated with active filler use. This response may be a combination of factors such as filling, water demand, hydration, and the timing of emulsion breaking; however, these mechanisms should be regarded as possible explanations and not as confirmed mechanisms unless backed by microstructural and chemical analysis.

### 3.2. Voids in Total Mix

**Figure 7:**
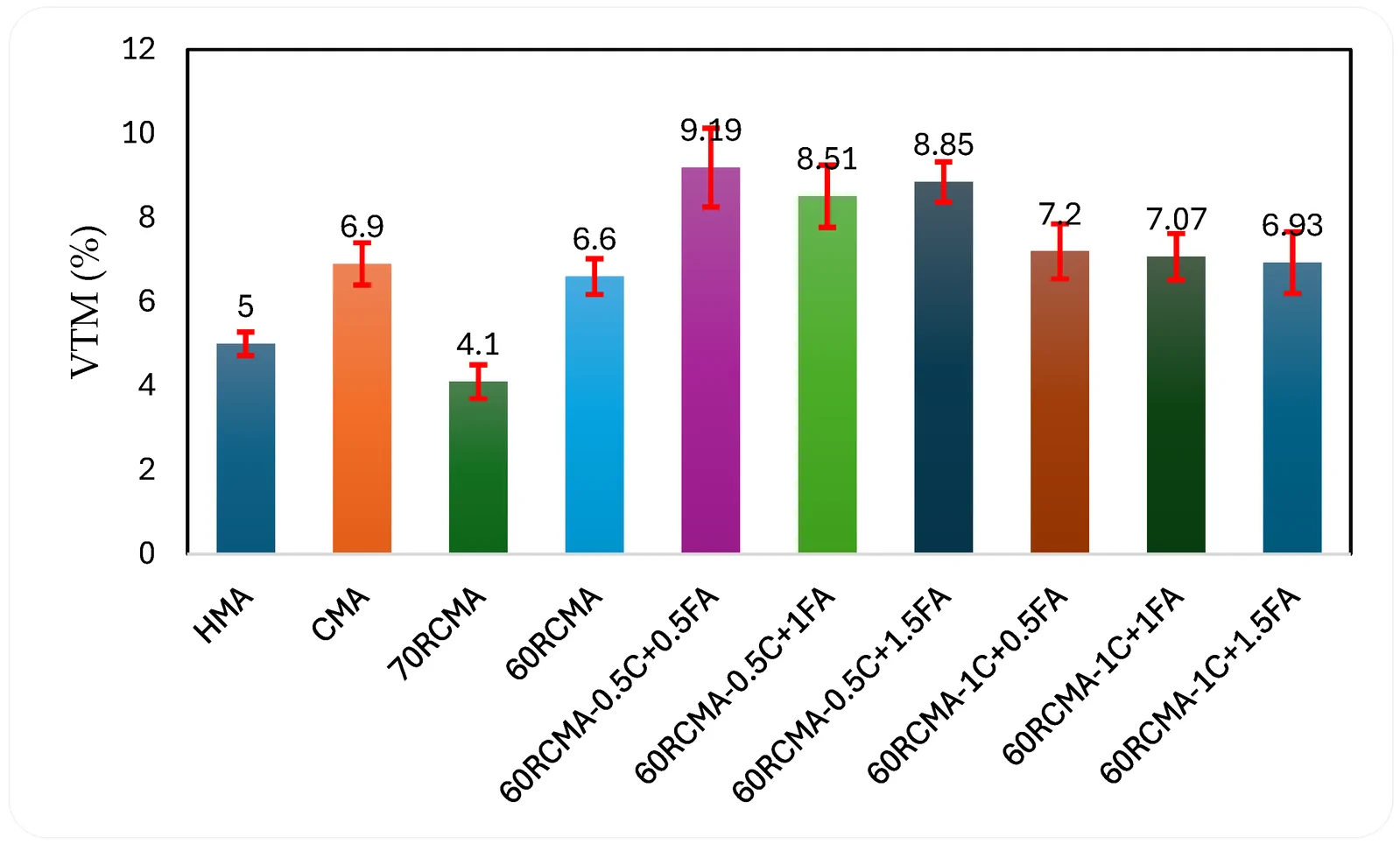
VTM results.

The VTM values reported in Figure 7 show the same trend as the density results above. However, care should be taken when comparing VTM values because it is not truly the inverse of bulk density since VTM is also a function of Gmm. The cold mixtures possessed the lowest VTM values with the 70RCMA mixture having a VTM value equal to 4.10%. For the CMA and 60RCMA mixtures, the VTM values were equal to 6.90% and 6.60%, respectively. Although 70RCMA showed a lower bulk density than HMA 2.25 vs. 2.36 g/cc, it nonetheless recorded a lower VTM, 4.1% vs. 5.0%. This apparent discrepancy reflects the fact that the VTM depends on bulk density relative to each mixture’s own theoretical maximum specific gravity, which differs with composition; a direct comparison of bulk density values across mixtures with differing material compositions therefore does not, on its own, indicate relative void content. It should also be noted that the comparatively low VTM of 70RCMA does not indicate a superior mixture, given that this formulation did not satisfy the target dense-graded specification envelope.

Furthermore, all the cement–fly ash-modified mixtures possessed higher VTM values than the unmodified 60RCMA mixture, indicating that the addition of active filler created an open structure initially. The VTM at 0.5% cement ranged from 8.51% to 9.19% with no clear trend on the effect of increasing fly ash content. Fixed 1% cement dosage and fly ash increment resulted in a gradual reduction in the VTM from 7.20% (0.5% fly ash) to 6.93% (1.5% fly ash). In contrast, altering cement content from 0.5% to 1.0% at a fixed fly ash dosage produced a substantial reduction in the VTM across all three fly ash levels (e.g., from 9.19% to 7.20% at 0.5% fly ash). These numerically observed trends indicate that cement dosage exerted a considerably stronger influence on compaction behavior than fly ash dosage. Once again, the result of this volumetric response coincides with that of the density results, suggesting that the higher cement dosage contributed to improved compaction density in the modified mixtures. The VTM and density were not evaluated statistically; therefore, this is reported as an observed trend rather than a confirmed effect. Among the tested mixtures, 60RCMA-1C + 1.5FA achieved the best density−VTM relationship.

### 3.3. Marshall Stability

**Figure 8:**
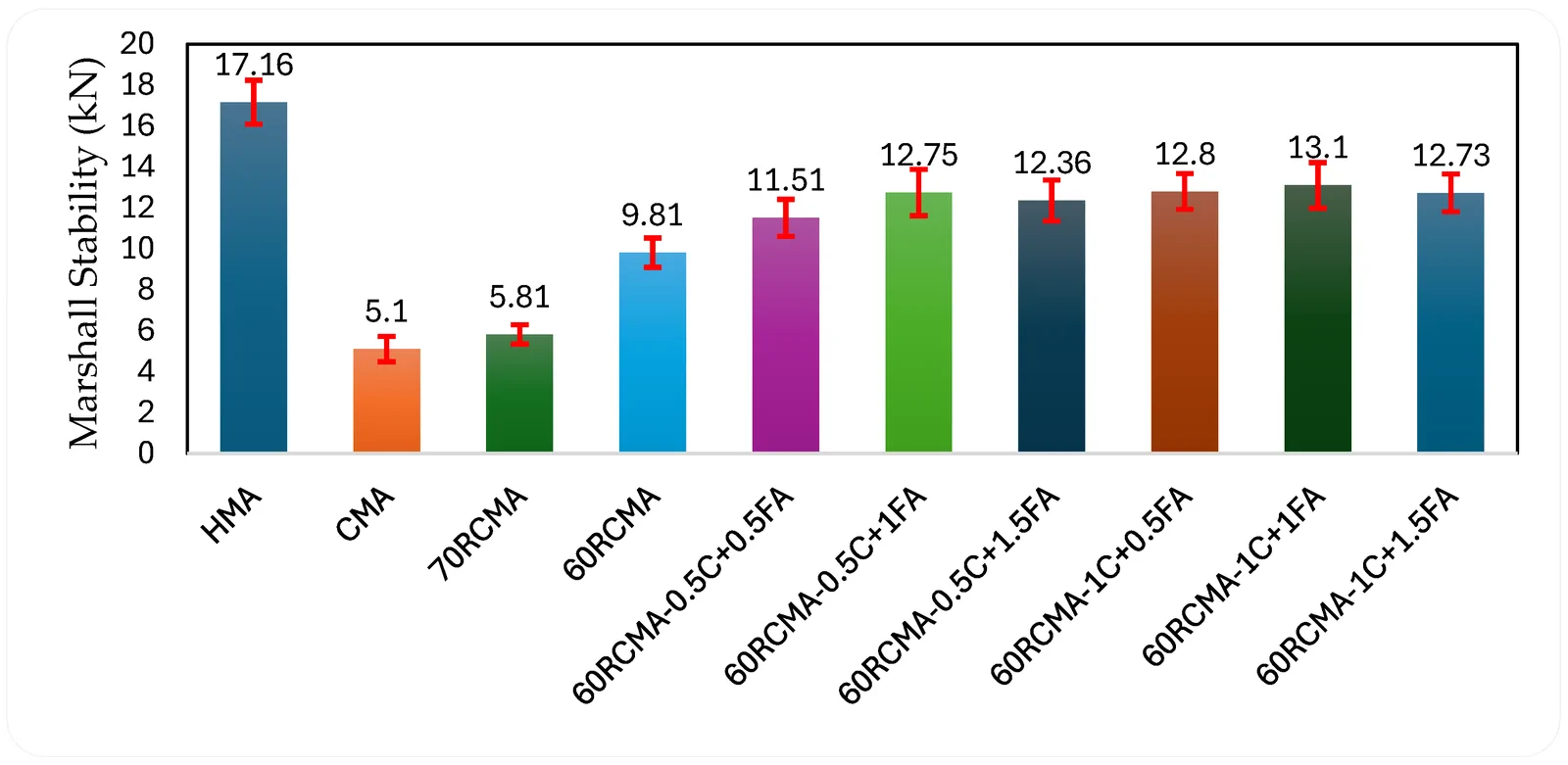
Marshall stability results.

Figure 8 represents Marshall stability. HMA produced 17.16 kN, whereas CMA without RAP produced 5.10 kN. The 70% RAP screening blend produced 5.81 kN and was outside the adopted gradation envelope. The 60% RAP blend produced 9.81 kN and was therefore used as the additive-stage base mixture. The six cement–fly ash formulations produced 11.51–13.10 kN, with the highest measured value at 1% cement + 1% fly ash. The improvement is consistent with better aggregate packing at the selected gradation and with accelerated emulsion breaking/hydration from the active additives. Overall, these results match existing studies [35,44].

### 3.4. Indirect Tensile Strength

**Figure 9:**
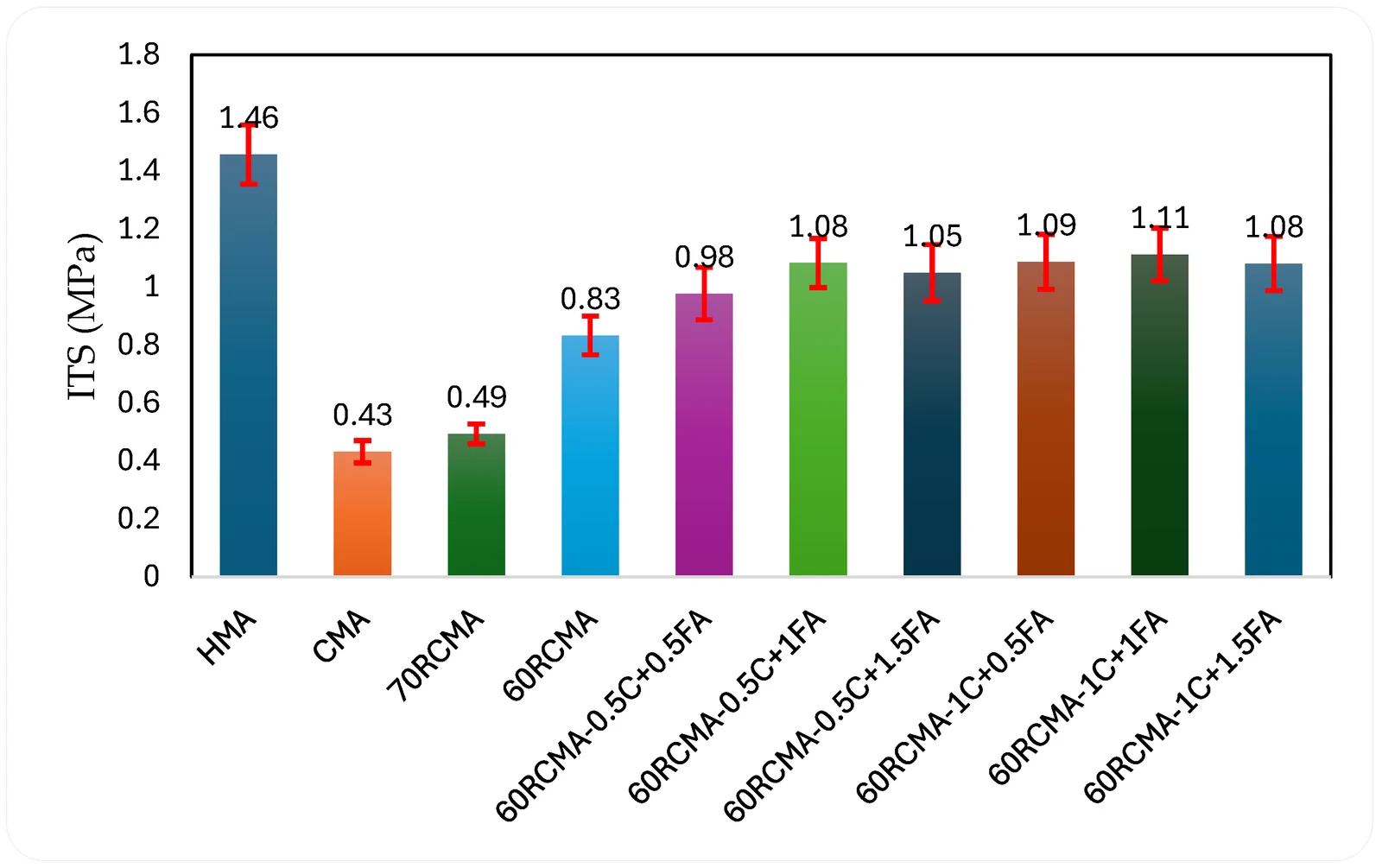
Dry ITS results.

Figure 9 presents the mean dry ITS. HMA produced 1.457 MPa and CMA without RAP produced 0.432 MPa. The 70% RAP screening blend produced 0.493 MPa, while the envelope-compliant 60% RAP blend produced 0.833 MPa. The additive mixtures produced 0.977–1.112 MPa; the highest measured value occurred at 1% cement + 1% fly ash. A denser load-bearing skeleton and hydration-assisted interfacial bonding plausibly contributed to the increase, whereas incomplete emulsion curing and higher void content could depress ITS. Overall, the ITS results are consistent with similar studies [35,44].

### 3.5. Marshall Quotient

**Figure 10:**
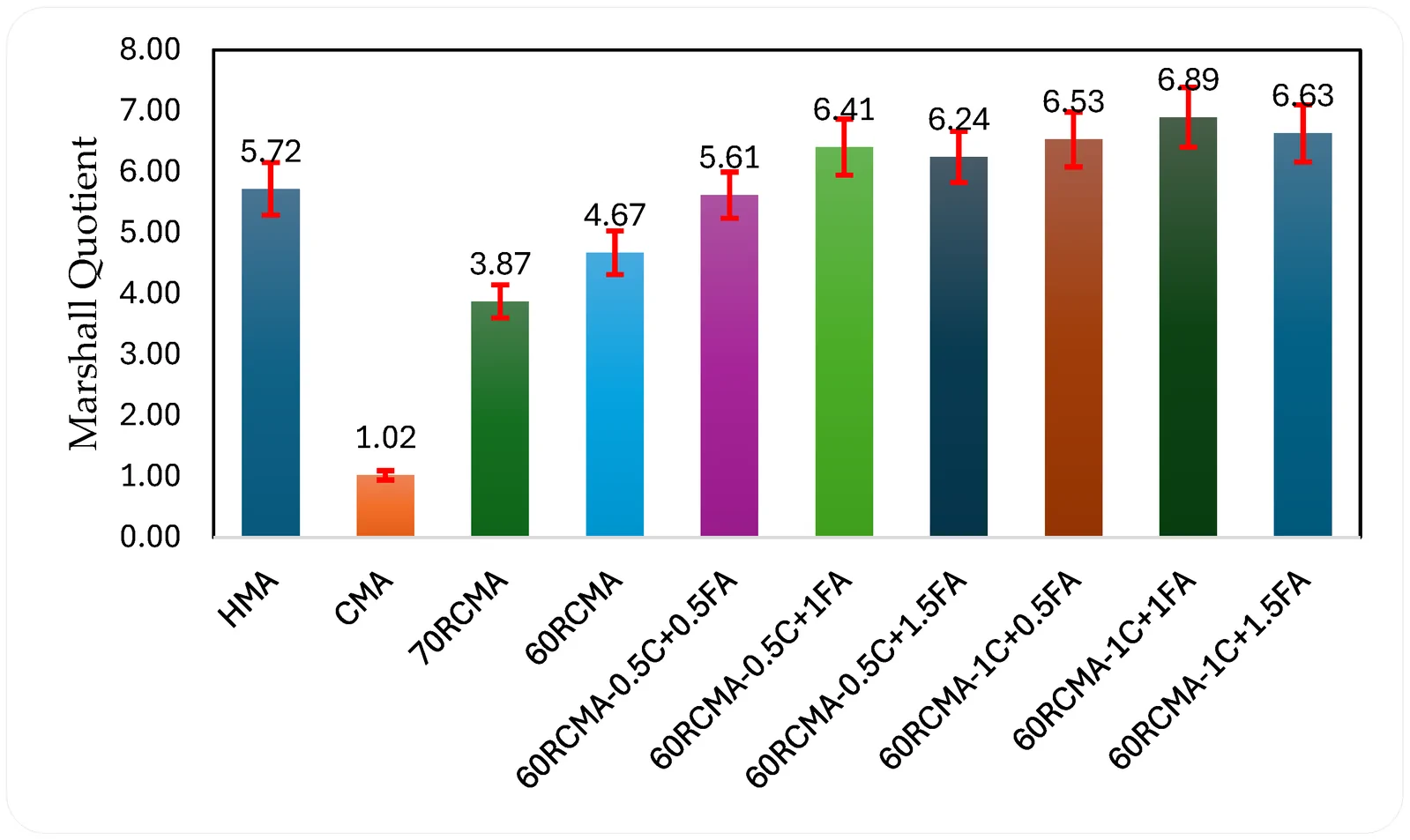
Marshall quotients of various mixes.

Figure 10 presents the mean MQ. The values were 5.72 for HMA, 1.02 for CMA, 3.87 for the 70% RAP screening blend, and 4.67 for the selected 60% RAP base mixture. The additive formulations produced 5.61–6.89, with the highest measured value at 1% cement + 1% fly ash. These results indicate increased resistance to deformation in the Marshall loading configuration, but they do not replace wheel-tracking or repeated-load permanent deformation testing.

### 3.6. Durability

**Figure 11:**
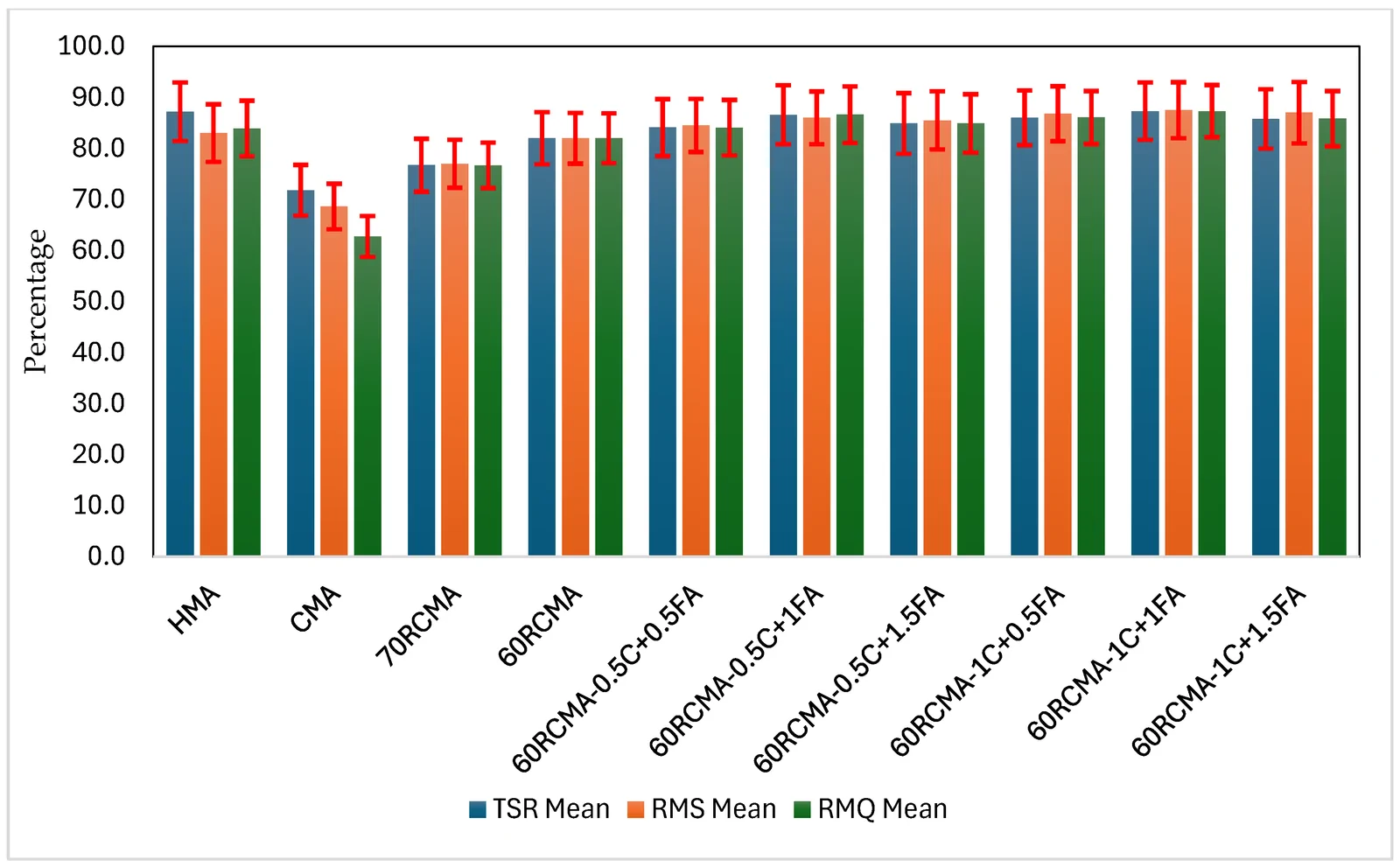
Durability results.

Durability was evaluated using TSR, RMS, and RMQ. The results are compared in Figure 11 with blue, red, and green bars, respectively.

These indices describe retained short-term properties under the stated conditioning schedules and are not a complete durability assessment.

TSR values were 87.2% for HMA, 71.8% for CMA, 76.7% for the 70% RAP screening blend, and 82.0% for the selected 60% RAP base mixture. The six additive formulations ranged from 83.2% to 87.3%, with the highest measured value at 1% cement + 1% fly ash. The lower retained strength of the unmodified cold mixtures is consistent with incomplete emulsion film development and moisture-sensitive interfaces. Hydration products and faster emulsion breaking may improve retained strength in the additive mixtures, but this mechanism requires direct microstructural verification.

RMS values followed the same broad pattern: 83.0% for HMA, 68.6% for CMA, 77.0% for the 70% RAP screening blend, 82.0% for the 60% RAP base mixture, and 84.1–87.5% for the additive formulations. The maximum measured RMS occurred at 1% cement + 1% fly ash.

RMQ values were 83.9% for HMA, 62.7% for CMA, 76.7% for the 70% RAP screening blend, 82.0% for the 60% RAP base mixture, and 84.1–87.3% for the additive formulations. The maximum measured RMQ again occurred at 1% cement + 1% fly ash.

Taken together, the three retained-property indices show numerically higher values for the modified formulations under the reported short-term conditioning. However, it was observed from the ANOVA results that neither the presence of filler modification nor cement and fly ash dosage had a statistically significant effect on the durability indices TSR, RMS, and RMQ. Additionally, these indices do not establish long-term resistance to fatigue, shrinkage cracking, thermal cracking, permanent deformation, or field aging. Cement production also carries an environmental burden, and coal fly ash availability may decline in some regions; therefore, environmental and economic benefits require a project-specific assessment.

The RMS red bars in Figure 11 follow trends similar to those discussed for TSR. Mix0 achieved an RMS value of 83.0%, Mix1 dropped sharply to 68.6%, and Mix2 improved to 77.0%. The optimized 60% RAP mix restored RMS to 82.0%. The incorporation of binary binders further enhanced RMS values to the range of 84.1–87.5%, with the highest value achieved by the 1% cement and 1% fly ash mixture. These results highlight the combined benefits of optimized gradation and limited cementitious modification in improving moisture resistance.

Concerning the RMQ results in Figure 10, Mix0 achieved 83.9%, Mix1 62.7%, and Mix2 76.7%. The optimized 60% RAP mix achieved an RMQ of 82.0, essentially matching Mix0. Binary binder modification further increased RMQ values to the range of 84.1–87.3, with the highest value recorded for the 1% cement + 1% fly ash mixture.

The consistent improvements across TSR, RMS, and RMQ confirm the effectiveness of binary binder systems in enhancing the durability and moisture resistance of RCMA. These results are consistent with existing studies [35,44].

### 3.7. Statistical Analysis with ANOVA

To evaluate the statistical significance of the observed trends, three separate ANOVA analyses were conducted, as described in Section 2.8.

(1) Effect of RAP Content

One-way ANOVA was employed to compare 0% RAP CMA, 60% RAP, and 70% RAP RCMA to analyze the effects of RAP content on MS, ITS, MQ, RMS, RMQ, and TSR. It was found that RAP content had a significant effect on these properties except TSR (Table 12). Tukey HSD post hoc comparison indicated that 60RCMA was significantly higher in MS and ITS than both CMA and 70RCMA, whereas CMA and 70RCMA were not significantly different, in agreement with the gradation non-compliance of the 70% RAP blend limiting effective binder–aggregate bonding despite its higher RAP-derived binder content. MQ, however, was significantly different among all three RAP levels, indicating that stiffness development is more sensitive to RAP content than stability or ITS individually. The absence of a significant RAP effect on TSR suggests that the aged RAP binder film did not significantly affect moisture susceptibility relative to unmodified CMA within the tested RAP range, despite the numerical improvement observed. For RMS and RMQ, only the CMA-to-60RCMA comparison was significant, following the same pattern as observed for MS and ITS.

**Table 12:** ANOVA results for RAP content (comparing Mix1, Mix2, and Mix3).

Property	SS	DF	MS	F	*p*-Value	Decision (α = 0.05)	Tukey HSD Interpretation
MS	38.688200	2, 6	19.344100	51.565932	0.000166	Significant	60RCMA > CMA and 70RCMA; CMA ≈ 70RCMA
ITS	0.279443	2, 6	0.139721	58.643473	0.000115	Significant	60RCMA > CMA and 70RCMA; CMA ≈ 70RCMA
MQ	22.085000	2, 6	11.042500	159.171171	0.000006	Significant	All three RAP levels significantly different
TSR	156.140000	2, 6	78.070000	3.008586	0.124465	Not significant	No pairwise differences
RMS	273.881400	2, 6	136.940700	6.157553	0.035158	Significant	60RCMA > CMA; other comparisons not significant
RMQ	592.418600	2, 6	296.209300	14.849519	0.004748	Significant	60RCMA and 70RCMA > CMA; 60RCMA ≈ 70RCMA

(2) Effect of Cement–Fly Ash Modification on 60RCMA

One-way ANOVA with Dunnett’s post hoc comparison, employed to compare six cement–fly ash-modified formulations with unmodified 60RCMA (Table 13), showed significant effects of filler modification on MS, ITS, and MQ for all formulations except 60RCMA-0.5C + 0.5FA. This indicates a minimum active filler dosage beyond which cementitious filler contributes to the strength gain mechanism. However, no significant filler modification effects were observed on the TSR, RMS, and RMQ values.

**Table 13:** ANOVA results for evaluating filler modification effects on unmodified 60RCMA and cement–fly ash-modified 60RCMA (comparing Mix3, Mix4 and Mix5).

Property	SS	DF	MS	F	*p*-Value	Decision (α = 0.05)	Dunnett’s Comparison vs. 60RCMA
MS	23.852057	6, 14	3.975343	4.304182	0.011486	Significant	All modified mixes except 0.5C + 0.5FA significantly higher
ITS	0.171888	6, 14	0.028648	3.637650	0.021788	Significant	All modified mixes except 0.5C + 0.5FA significantly higher
MQ	10.438200	6, 14	1.739700	9.187401	0.000341	Significant	All modified mixes except 0.5C + 0.5FA significantly higher
TSR	56.691429	6, 14	9.448571	0.299707	0.926827	Not significant	No modified mix differed significantly
RMS	64.045714	6, 14	10.674286	0.363088	0.890308	Not significant	No modified mix differed significantly
RMQ	57.731829	6, 14	9.621971	0.338320	0.905202	Not significant	No modified mix differed significantly

(3) Effects of Cement–Fly Ash Dosages

Two-way ANOVA on six cement–fly ash formulations (Table 14) indicated that cement dosage has a significant effect only on MQ, with the 1% cement level producing significantly higher MQ than the 0.5% level, which is consistent with cement’s role in accelerating hydration-based stiffness development. Fly ash dosage and the cement × fly ash interaction had no statistically significant effect on any evaluated property. Although 60RCMA-1C + 1FA showed higher numerical responses across evaluated properties, the factorial analysis does not support this formulation as superior to the others. However, cement dosage at the 1% level appears to be the factor responsible for stiffness gain within the modified group.

**Table 14:** Two-way ANOVA results for cement and fly ash dosage effects (cement at 0.5%/1.0%; fly ash at 0.5%/1.0%/1.5%) (comparing Mix4 and Mix5).

Property	Cement F(1, 12)	Cement *p*	Fly Ash F(2, 12)	Fly Ash *p*	Cement × FA F(2, 12)	Interaction *p*	Significant Factor (s)
MS	2.04	0.179	0.90	0.434	0.44	0.656	None
ITS	1.73	0.213	0.76	0.488	0.37	0.701	None
MQ	8.04	0.015	2.59	0.116	0.61	0.562	Cement only
TSR	0.19	0.671	0.19	0.827	0.02	0.981	None
RMS	0.47	0.508	0.06	0.942	0.01	0.989	None
RMQ	0.22	0.646	0.21	0.810	0.02	0.976	None

## 4. Conclusions

This study screened two high-RAP blends within a dense-graded cold-mix framework and evaluated six cement–Class C fly ash formulations at the selected 60% RAP level using HMA as a benchmark. The conclusions are restricted to the materials, gradation envelope, curing regime, and short-term tests used:

1. HMA exhibited the highest mechanical performance among all mixtures, owing to better compaction and binder–aggregate bonding under hot-mix conditions. However, unmodified CMA showed poor mechanical response due to the relatively slow curing behavior of emulsion-based binder compared to HMA.
2. RAP incorporation significantly improved MS, ITS, MQ, RMS, and RMQ (one-way ANOVA, *p* < 0.05), but not TSR (*p* = 0.124). The 60% RAP formulation (60RCMA) achieved significantly higher stability and ITS than both unmodified CMA and 70RCMA, while 70RCMA, which fell outside the adopted dense-graded envelope, did not differ significantly from CMA despite its higher RAP-derived binder content. This indicates that gradation envelope compliance, rather than RAP content alone, governed short-term strength development in this system; 60% RAP therefore represents the highest RAP content feasible within the adopted dense HMA-type gradation framework.
3. Cement–fly ash modification of 60RCMA produced statistically significant increases in MS, ITS, and MQ (one-way ANOVA with Dunnett’s comparison, *p* < 0.05) relative to the unmodified mixture, with all six modified formulations except 60RCMA-0.5C + 0.5FA differing significantly from the control. However, for TSR, RMS, and RMQ, no significant modification effect was observed but all these remained about 87%. Among the modified formulations, 60RCMA1C + 1FA showed the highest numerical stability, ITS, and MQ values (13.1 kN, 1.11 MPa, and 6.89, respectively). The improvement is consistent with combined effects of aggregate packing, emulsion breaking, and cementitious bonding, but those mechanisms require chemical and microstructural confirmation. Also, it was observed in two-way ANOVA analysis that this formulation was not statistically different from most other formulations. The only factor that remained significant was cement dosage, and only at the 1% level, affecting MQ only (*p* = 0.015). It further showed that cement–fly ash dosages and their interaction had no statistically significant effect on any moisture-related index (TSR, RMS, or RMQ).
4. These results revealed that 60% RAP modified with cement–fly ash demonstrated improved short-term mechanical performance and maintained sufficient moisture resistance under the limited laboratory testing conditions, materials, and gradation envelope. Since Marshall stability, ITS, MQ, TSR, RMS, and RMQ are short-term screening indicators, these results do not describe fatigue behavior, permanent deformation, or long-term durability, especially considering the potential trade-off of increased hydraulic bonding with mixture flexibility. Further work should include microstructural characterization using SEM, FTIR, and XRD to verify the proposed reaction mechanisms, dynamic mechanical testing (e.g., fatigue, dynamic modulus, and wheel-tracking) to assess long-term performance, and a life-cycle and economic assessment to quantify the environmental and cost implications of cement and fly ash use, including the associated CO_2_ emissions of cement and the regional variability of fly ash.

## Figures and Tables

**Table 1:** Physical properties of limestone aggregate.

Property	Unit	Standard	Limit	Results
**Coarse aggregates (5–25 mm)**				
Aggregate impact value	%	IS:2386 Part IV	≤30	22.3
Los Angeles abrasion value	%	AASHTO T96	≤30	21
Flakiness index	%	IS:2386 Part I	25–30	26.7
Elongation index	%	IS:2386 Part I	25–30	28
Crushing strength	%	IS:2386 Part IV	≤30	18
Coarse aggregate (bulk) specific gravity	g/cm^3^	AASHTO T85	2.5–3.0	2.62
Coarse aggregate (app) specific gravity	g/cm^3^	AASHTO T84	>2.6	2.63
**Fine aggregates (0–5 mm)**				
Fine-aggregate specific gravity	g/cm^3^	AASHTO T84	2.5–2.9	2.62
Water absorption	%	ASTM C127/C128	≤2	1.17

**Table 2:** Physical properties of bitumen.

Property	Unit	Standard	Limit	Results
Penetration test at 25 °C	mm	AASHTO T49	60–70	65.3
Ductility test at 25 °C	cm	AASHTO T51	≥100	134
Softening point test	°C	AASHTO T53	45–55	45
Specific gravity test	g/cm^3^	ASTM D70	1.01–1.06	1.02
Flash point test	°C	ASTM D92	≥232	319
Fire point test	°C	ASTM D92	≥250	363

**Table 3:** Physical properties of cationic slow-setting emulsion (CSS-1h).

Property	Unit	Standard	Limit	Results
Viscosity, Saybolt-Furol, 25 °C SFS		ASTM D7496	20–100	24
Particle charge test		ASTM D7402	-	Positive
Specific Gravity	g/cm^3^	ASTM D6937	0.95–1.10	1.0185
Residue by distillation	%	ASTM D6997	≥57	60
Penetration, 25 °C, 100 gm, 5 s	mm	ASTM D5	40–90	62
Ductility, 25 °C, 5 cm/min	cm	ASTM D113	≥40	100
Solubility in trichloroethylene	%	ASTM D2042	≥97.5	98.5

**Table 4:** Physical properties of RAP and extracted bitumen from RAP.

Property	Unit	Standard	Limit	Results
Specific gravity of RAP	g/cm^3^	AASHTO T85	2.4–2.7	2.498
Specific gravity of extracted bitumen at 25 °C	g/cm^3^	AASHTO T228	1.01–1.05	1.04
Bitumen content	%	AASHTO T164 (extraction)/AASHTO T308 (ignition)	≥2	2.5
Penetration at 25 °C	0.1 mm	AASHTO T49	20–70	55
Softening point	°C	AASHTO T53	50–65	58
Ductility at 25 °C	cm	AASHTO T51	≥50	101

**Table 5:** RAP gradation.

Sieve Size		RAP Aggregate Gradation
Inch	mm	
1″	25.4	100
3/4″	19.1	84.8
1/2″	12.7	45.5
3/8″	9.52	28.6
#4	4.76	7.0
#8	2.40	1.9
#50	0.30	0.6
#200	0.075	0.3

**Table 6:** Physical and chemical properties of ordinary Portland cement.

Properties of OPC	Results
**Physical Property**	
Specific gravity of OPC	3.11
**Chemical Properties**	
Potassium oxide, K_2_O	0.66%
Sodium oxide, Na_2_O	0.24%
Sulfite [SO_3_]	2.85%
Magnesium oxide [MgO]	2.2%
Ferric oxide [Fe_2_O_3_]	3.41%
Aluminum oxide [Al_2_O_3_]	6.4%
Silicon dioxide [SiO_2_]	20.6%
Calcium oxide [CaO]	62.23%
Loss on ignition	1.61%

**Table 7:** Physical and chemical properties of Type-C fly ash.

Properties of Class C Fly Ash	Results
**Physical Property**	
Specific gravity of fly ash	2.71
**Chemical Properties**	
Sodium oxide, Na_2_O	1.34%
Sulfite, SO_3_	1.85%
Magnesium oxide, MgO	7.2%
Ferric oxide, Fe_2_O_3_	3.41%
Aluminum oxide, Al_2_O_3_	19.4%
Silicon dioxide, SiO_2_	47.6%
Calcium oxide, CaO	21%

**Table 8:** Designation of mixtures.

Mix Designation	Mix Description
Mix0	Control HMA with 0% RAP (*HMA*)
Mix1	Control CMA with 0% RAP (*CMA*)
Mix2	CMA with 70% RAP (*70RCMA*)
Mix3	CMA with 60% RAP (*60RCMA*)
Mix4	CMA with 60% RAP modified with 0.5% OPC and fly ash 0.5%, 1%, and 1.5% (*60RCMA-0.5C + 0.5FA, 60RCMA-0.5C + 1FA, 60RCMA-0.5C + 1.5FA*)
Mix5	CMA with 60% RAP modified with 1% OPC and fly ash 0.5%, 1%, and 1.5% (*60RCMA-1C + 0.5FA, 60RCMA-1C + 1FA, 60RCMA-1C + 1.5FA*)

**Table 9:** Aggregate blend for 70% and 60% RAP RCMA.

Sieve Size				Percentage Passing %	
Inch	mm	70% RAP	60% RAP	Midpoint Specification	Specification Limit
1″	25.4	100.0	100.0	100	100
3/4″	19.1	89.4	90.9	95	90- 100
1/2″	12.7	61.9	67.3	---	---
3/8″	9.52	49.8	56.7	63	56–70
#4	4.76	33.9	41.5	42.5	35–50
#8	2.4	24.3	30.5	29	23–35
#50	0.3	6.4	8.0	8.5	5–12
#200	0.075	2.3	2.9	5	2–8

**Table 10:** Calculated values of IRAC, IEC, Pnb and optimum Pnb, optimum total liquid content, and optimum residual asphalt.

Mix	IRAC (%)	RAP (%)	r (%)	Pnb (%)	IEC (%)	OPWWC (%)	OTLC (%)	Optimum (OPnb) (%)	RAP Binder Contribution in (ORAC) (%)	ORAC (%)
Control CMA	6.11	0	100	--	10.18	4.0	12.18	6.3	--	6.3
60RCMA	5.10	60	40	3.6	6.0	3.5	7.5	3.766	1.5	5.3
70RCMA	4.70	70	30	2.95	5.25	3.0	6.92	3.15	1.75	4.9

**Table 11:** Complete formulation matrix (percent by mass of total dry aggregate).

Mix	5–12 mm (g/%)	0–5 mm (g/%)	RAP (g/%)	Dry Agg. Total	Cement (g/%)	Fly Ash (g/%)	Emulsion (g/%)	Added Water (g/%)	OTLC (g/%)	Final Mix Mass
HMA	—	—	—	1149.24	—	—	50.76 (4.23%) bitumen	—	—	1200
CMA	—	—	—	1200	—	—	126 (10.50%)	20.16 (1.68%)	146.16 (12.18%)	1346.16
70RCMA	12 (1%)	348 (29%)	840 (70%)	1200	—	—	63 (5.25%)	20.04 (1.67%)	83.04 (6.92%)	1283.04
60RCMA	36 (3%)	444 (37%)	720 (60%)	1200	—	—	75.36 (6.28%)	14.68 (1.22%)	90 (7.5%)	1290
60RCMA-0.5C + 0.5FA	36 (3%)	432 (36%)	720 (60%)	1200	6.0 (0.5%)	6.0 (0.5%)	75.36 (6.28%)	14.68 (1.22%)	90 (7.5%)	1290
60RCMA-0.5C + 1FA	36 (3%)	426 (35.5%)	720 (60%)	1200	6.0 (0.5%)	12.0 (1.0%)	75.36 (6.28%)	14.68 (1.22%)	90 (7.5%)	1290
60RCMA-0.5C + 1.5FA	36 (3%)	420 (35%)	720 (60%)	1200	6.0 (0.5%)	18.0 (1.5%)	75.36 (6.28%)	14.68 (1.22%)	90 (7.5%)	1290
60RCMA-1C + 0.5FA	36 (3%)	426 (35.5%)	720 (60%)	1200	12.0 (1.0%)	6.0 (0.5%)	75.36 (6.28%)	14.68 (1.22%)	90 (7.5%)	1290
60RCMA-1C + 1FA	36 (3%)	420 (35%)	720 (60%)	1200	12.0 (1.0%)	12.0 (1.0%)	75.36 (6.28%)	14.68 (1.22%)	90 (7.5%)	1290
60RCMA-1C + 1.5FA	36 (3%)	414 (34.5%)	720 (60%)	1200	12.0 (1.0%)	18.0 (1.5%)	75.36 (6.28%)	14.68 (1.22%)	90 (7.5%)	1290

## Data Availability

The original contributions presented in this study are included in the article. Further inquiries can be directed to the corresponding authors.

## Abbreviations

The following abbreviations are used in this manuscript:

HMA	Hot-mix asphalt
CMA	Cold-mix asphalt without RAP
RCMA	Recycled cold-mix asphalt with RAP
70RCMA	Recycled cold-mix asphalt with 70% RAP
60RCMA	Recycled cold-mix asphalt with 60% RAP
60RCMA-0.5C + 0.5FA	Recycled cold-mix asphalt with 60% RAP modified with 0.5% cement and 0.5% fly ash
60RCMA-0.5C + 1FA	Recycled cold-mix asphalt with 60% RAP modified with 0.5% cement and 1% fly ash
60RCMA-0.5C + 1.5FA	Recycled cold-mix asphalt with 60% RAP modified with 0.5% cement and 1.5% fly ash
60RCMA-1C + 0.5FA	Recycled cold-mix asphalt with 60% RAP modified with 1% cement and 0.5% fly ash
60RCMA-1C + 1FA	Recycled cold-mix asphalt with 60% RAP modified with 1% cement and 1% fly ash
60RCMA-1C + 1.5FA	Recycled cold-mix asphalt with 60% RAP modified with 1% cement and 1.5% fly ash
ANOVA	Analysis of variance
RAP	Reclaimed asphalt pavement
CSS-1h	Cationic slow-setting asphalt emulsion with lower viscosity and harder base bitumen
FA	Fly ash
OPC	Ordinary Portland cement
MS	Marshall stability
ITS	Indirect tensile strength
TSR	Tensile strength ratio
MQ	Marshall quotient
RMQ	Retained Marshall quotient
RMS	Retained Marshall stability
IRAC	Initial residual asphalt content
IEC	Initial emulsion content
OBC	Optimum bitumen content
OEC	Optimum emulsion content
OFC	Optimum filler content
OPWwc	Optimum pre-wetted water content
ORAC	Optimum residual asphalt content
OTLC	Optimum total liquid content
RAC	Residual asphalt content
